# Activation of the Nlrp3 Inflammasome Contributes to Shiga Toxin-Induced Hemolytic Uremic Syndrome in a Mouse Model

**DOI:** 10.3389/fimmu.2020.619096

**Published:** 2021-01-21

**Authors:** Liqiong Song, Yuchun Xiao, Xianping Li, Yuanming Huang, Guangxun Meng, Zhihong Ren

**Affiliations:** ^1^State Key Laboratory for Infectious Disease Prevention and Control, National Institute for Communicable Disease Control and Prevention, Chinese Center for Disease Control and Prevention, Research Units of Discovery of Unknown Bacteria and Function (2018 RU010), Chinese Academy of Medical Sciences, Beijing, China; ^2^The Center for Microbes, Development and Health, CAS Key Laboratory of Molecular Virology & Immunology, Chinese Academy of Sciences, Institute Pasteur of Shanghai, University of Chinese Academy of Sciences, Shanghai, China

**Keywords:** Stx2, hemolytic uremic syndrome, IL-1β, Nlrp3, Nlrp3 inhibitor

## Abstract

**Objective:**

To explore the role of the Nlrp3 inflammasome activation in the development of hemolytic uremic syndrome (HUS) induced by Stx2 and evaluate the efficacy of small molecule Nlrp3 inhibitors in preventing the HUS.

**Methods:**

Peritoneal macrophages (PMs) isolated from wild-type (WT) C57BL/6J mice and gene knockout mice (*Nlrc4*^-/-^, *Aim2*^-/-^, and *Nlrp3*^-/-^) were treated with Stx2 *in vitro* and their IL-1β releases were measured. WT mice and *Nlrp3*^-/-^ mice were also treated with Stx2 *in vivo* by injection, and the biochemical indices (serum IL-1β, creatinine [CRE] and blood urea nitrogen [BUN]), renal injury, and animal survival were compared. To evaluate the effect of the Nlrp3 inhibitors in preventing HUS, WT mice were pretreated with different Nlrp3 inhibitors (MCC950, CY-09, Oridonin) before Stx2 treatment, and their biochemical indices and survival were compared with the WT mice without inhibitor pretreatment.

**Results:**

When PMs were stimulated by Stx2 *in vitro*, IL-1β release in *Nlrp3*^-/-^ PMs was significantly lower compared to the other PMs. The *Nlrp3*^-/-^ mice treated by Stx2 *in vivo*, showed lower levels of the biochemical indices, alleviated renal injuries, and increased survival rate. When the WT mice were pretreated with the Nlrp3 inhibitors, both the biochemical indices and survival were significantly improved compared to those without inhibitor pretreatment, with Oridonin being most potent.

**Conclusion:**

Nlrp3 inflammasome activation plays a vital role in the HUS development when mice are challenged by Stx2, and Oridonin is effective in preventing HUS.

## Introduction

Escherichia coli (*E. coli*) O157: H7 and *E. coli* O104: H4 pose a serious concern worldwide because they can injure intestinal mucosa and erythrocytes leading to hemorrhagic enteritis and hemolytic uremic syndrome (HUS) in humans or animals (1, 2). In 1993, 501 people were infected with *E. coli* O157: H7 from eating contaminated beef in the United States, which resulted in 45 people developing HUS, and three children died (3). In 1996, consumption of *E. coli* O157: H7 contaminated-radish seedlings caused hemorrhagic enteritis epidemic in Osaka, Japan (4). O157: H7 outbreaks also occurred in China with more than 20,000 infections, 195 HUSs, and 177 deaths in 1999 (5). An outbreak of *E. coli* O104: H4 in northern Germany in 2011 also led to more than 3222 infections and 32 deaths (6).

Acute HUS often manifests as hemolytic anemia, thrombocytopenia, and acute renal failure. The death or end-stage renal disease occurred with a pooled incidence of 12% and 25% of survivors demonstrated long-term renal sequelae (7). Several studies have shown that Shiga toxin (Stx) is the key virulence factor in developing acute HUS (2, 8, 9). Both Stx1 and Stx2 are cytotoxic to Vero cell (10) and they share a common conserved structure consisting of one biologically active A subunit associated with five identical B subunits that allow binding of the toxin to the globotriaosylceramide (Gb3) receptor. When being transferred into the cytoplasm, subunit A has RNA N-glycosidase activity and inhibits protein synthesis by removing an adenine nucleotide from 28 S rRNA of the 60S large subunit of the ribosome (11, 12). Although the ability of Stx1 to bind to receptor Gb3 is stronger than that of Stx2, several studies have revealed that Stx2 has stronger toxicity than Stx1 (13). The toxicity of Stx2 to human renal microvascular endothelial cells is 1,000 times stronger than that of Stx1 (14). Some studies have also shown that, compared with Stx1, Stx2 has a stronger correlation with hemorrhagic enteritis or HUS (15). Stx1 mainly targeted the lungs, while the Stx2 primarily targeted the kidneys (16). Therefore, most of the studies are mainly focused on Stx2 and Stx2-targeted drugs, such as neutralizing antibodies and small molecule inhibitors when exploring the mechanism and therapeutic strategy.

It was reported that serum levels of pro-inflammatory cytokines such as IL-1β and TNF-α were significantly higher in HUS patients than in non-HUS patients, which suggested the critical role of the inflammatory response in HUS development (17, 18). Lee et al. demonstrated that Stx2 triggered the release of pro-inflammatory cytokines *via* Nlrp3 inflammasome activation and promoted caspase-8/3-dependent apoptosis in THP-1 cells (19). The increased levels of IL-1β and TNF-α, two pro-inflammatory cytokines, may be associated with disease severity. Ikeda M et al. has successfully established a mouse HUS model using Stx2 along with lipopolysaccharide (LPS) (17). Notably, the intraperitoneal (i.p.) administration of Stx2 alone failed to induce HUS development in a mouse model unless it is used in combination with LPS to induce the inflammatory response (17).

The inflammasomes play an essential role in the development of many diseases. Among them, the Nlrp3 inflammasome has been the one most thoroughly studied. Nlrp3 is an intracellular pattern recognition receptor that can be activated by sensing stimulus events from various pathogens to host signals. Activation of Nlrp3 results in cleavage of precursors of IL-1β and IL-18 into their mature forms and triggering of cell pyroptosis (20). The Nlrp3 inflammasome activation is associated with many diseases, including diseases of kidney, liver, lung, and central nervous system, and metabolic disorders such as diabetes type 2, atherosclerosis, obesity, gout (21). Platnich et al. suggest that Stx2/LPS compounds activate the production of mitochondrial reactive oxygen species (ROS), the upstream event of Nlrp3 inflammasome, thereby promoting pro-inflammatory cytokine maturation and pyroptosis *via* Nlrp3 inflammasome activation (22). Up to date, there is no evidence supporting that activation of Nlrp3 inflammasome contributes to the development of the Stx2/LPS-induced HUS in the *in vivo* condition. Therefore, we conducted the current study to test our hypothesis that Stx2/LPS induces the HUS by activating the Nlrp3 inflammasome.

Small molecule Nlrp3 inhibitors, such as MCC950, CY-09 and Oridonin, have shown the potential therapeutic effects in many Nlrp3-associated diseases. Five such inhibitors (MCC950, CY-09, OLT1177, Tranilast and Oridonin) have been shown to have good therapeutic potential by directly targeting the Nlrp3 proteins themselves, and specifically inhibiting Nlrp3 activation and thereby reducing IL-1β production (23). However, it is unknown whether these inhibitors have a therapeutic effect on Stx2/LPS-induced HUS. Here, using the Stx2/LPS-induced HUS mouse model, we tested whether the activation of the Nlrp3 inflammasome contributes to the development of Stx2/LPS-induced HUS and evaluated the therapeutic effect of specific Nlrp3 inhibitors in preventing HUS caused by Stx2 as a step of identifying new candidate drugs.

## Material and Methods

### Animal Welfare

All animal procedures were performed according to the protocols approved by the Laboratory Animal Welfare and Ethics Committee of the National Institute for Communicable Disease Control and Prevention, Chinese Center for Disease Prevention and Control. All procedures were performed in the Biosafety Level II laboratory and Animal Biosafety Level II laboratory.

### Preparation and Identification of Stx2 and Subunit B of Stx2

In this study, peritoneal macrophages (PMs) or mice were treated with holotoxin Stx2 or its negative control subunit B (Stx2B). The gene sequences of Stx2 and Stx2B were cloned into the expression plasmid pET32a after optimization of genetic codon to construct plasmid pET32a-Stx2 and pET32a-Stx2B respectively. These plasmids were then transferred into *E. coli* BL-21 (DE3) to express the proteins of Stx2 and Stx2B. The bacteria were cultured with 0.75mM Isopropyl β-D-Thiogalactoside (IPTG) at 37°C for 4 h and the cultures were collected by centrifugation at 12,000 rpm for 5 min. The resulting pellets were lysed by ultrasonication. The supernatant of the culture lysate was collected by centrifugation at 10,000 rpm, 4°C for 10 min. The two recombinant proteins of Stx2 and Stx2B with His-label were purified by protein purification instrument and two-step Ni column. The two recombinant proteins were analyzed by Western-blotting. Endotoxin was removed using De-toxi-Gel (Pierce Biotechnology) according to the manufacturer’s instructions. BCA kit was used to measure protein concentration (**Figure S1** in supplementary appendix). The same batches of recombinant Stx2 toxin and Stx2B protein were used throughout all the subsequent experiments in this study. The cytotoxicity of purified Stx2 and Stx2B was assessed using Vero cells (24).

### *In Vitro* Experiments

#### Cell Culture and Reagents

Mouse bone-marrow-derived macrophages (BMDMs) isolated from the wild-type (WT) C57BL/6J mice (female, eight weeks of age) and the genetically deficient mice were cultured as previously described (25). PMs were collected from peritoneal lavage according to the procedure reported by Kumagai (26). The purity of the macrophages was assessed by flow cytometry using the F4/80 antibody and shown to be over 90%. The differentiation of the THP-1 human monocytic cell line was achieved after incubation for 48 h in the presence of 10 nM phorbol myristate acetate (PMA, P8139, Sigma). All cultured cells were grown in RPMI 1640 at a maximum density of 1×10^6^ cells/ml.

#### Cytokine and Cytotoxicity Detection

PMs from the WT or genetically deficient mice (*Nlrc4*^-/-^, *Aim2*^-/-^ and *Nlrp3*^-/-^) were pretreated with 100 ng/ml LPS for 4 h for priming. Stx2 was incubated with the primed PMs in 24-well plates at a concentration of 2 µg/ml for 16 h after LPS was washed off with PBS. At the indicated time points, lactate dehydrogenase (LDH) activity in the culture supernatants as an indicator of the cytotoxicity of Stx2 was measured with a Cytotox96 Kit (Promega, Madison, WI) according to the manufacturer’s instructions. IL-1β and TNF-α in cell-free supernatants were quantified by ELISA kits according to the manufacturer’s protocols (BD Biosciences, San Jose, CA).

#### Western Blotting Analysis

The cultured supernatants and cell lysates were collected at the indicated time points after Stx2 treatment, and protein in the cell-free supernatants was concentrated using the methanol-chloroform precipitation method (27). The cell pellets were lysed with the RIPA Lysis buffer (89901, Thermo) supplemented with a 1:50 diluted protease inhibitor cocktail tablet (EDTA-free protease inhibitor cocktail tablet, Roche). Such prepared samples were then mixed with the equal volume of 2× SDS-loading buffer and detected for pro-IL-1β/IL-1β and pro-caspase-1/caspase-1 by immunoblotting. β-Actin was used as the positive control. Immobilized proteins were incubated with primary antibodies against IL-1β (sc-52012; 1:1,000), caspase-1 (AG-20B-0042; 1:1,000), and β-actin (4967S; 1:1,000), and followed by incubation with the secondary antibodies (IRDye 800-labeled anti-rabbit IgG; 611-132-002; 1:10,000 (Santa Cruz Biotechnology). The protein levels were detected using an Odyssey Infrared Imaging System (LI-COR, Lincoln, NE).

#### Blocking Nlrp3 Activation *In Vitro*

In the *in vitro* inhibiting experiment, LPS-primed PMs were pre-incubated with the following inhibitors: KCl (50 mM, PB0440, Sangon Biotech) to block K^+^ efflux, ATP receptor P2X7R inhibitor oxidized ATP (oATP, 500 μM, A6779, Sigma-Aldrich), cathepsin B inhibitor CA-074Me (10 μM, C5857, Calbiochem), Nlrp3 inhibitors including MCC950 (10 μM, S7809, Selleckchem), CY-09 (5 μM, S5774, Selleckchem) and Oridomin (20 μM, S2335, Selleckchem), and Caspase-1 inhibitor Z-YVAD-FMK (10 μM, A3707, Alexis Biochemicals), at the indicated concentrations for 1 h. Then, LPS-primed PMs were treated with the Stx2 for 16 h *in vitro*. All supernatants were collected and detected for IL-1β by ELISA and LDH with Cytotox 96 Kit.

#### *In Vivo* Experiments

##### Mice

All mice used in our experiments are based on a C57BL/6J genetic background, and all experiments were conducted with age- and gender-matched mice (8–10 weeks old, female). C57BL/6J wild-type (WT) mice were obtained from Beijing Vital River Laboratory Animal Technology Co. Ltd. To determine whether NLRP3 inflammasome is specifically required in the process of Stx2-induced IL-1β release *in vitro* and HUS development *in vivo*, we used Nlrp3-deficient (Nlrp3^-/-^), Aim2-deficient (Aim2^-/-^), and Nlrc4-deficient (Nlrc4^-/-^) mice in this study. provided Nlrp3^-/-^ mice were provided by Warren Strober at NIH, and Aim2^-/-^and Nlrc4^-/-^ mice were by Meng Guangxun at Institute Pasteur of Shanghai, Chinese Academy of Sciences (28–31). Only female mice at eight weeks of age were used in all the experiments.

#### Establishment of the Stx2/LPS-Induced Hemolytic Uremic Syndrome Mouse Model

To establish mouse HUS models, we followed the methods described by Ikeda et al. (17). Briefly, C57BL/6J mice were injected with Stx2 and LPS to induce HUS model. Mice in Stx2/LPS group as HUS model were injected intraperitoneally (i.p.) with 100 μl of Stx2 (2 µg/ml) on day 1 and 100 μl Stx2 (2 µg/ml) together with LPS (100 µg/ml) on day 2. The six mice in Stx2B/LPS group were injected i.p. with 100 μl of Stx2B (2 µg/ml) on day 1 and 100 μl of Stx2B (2 µg/ml) and LPS (100 µg/ml) on day 2. The different control group mice in PBS group, LPS group, Stx2 group or Stx2B group were injected i.p. with 100 μl of PBS, LPS (100 µg/ml), Stx2 (2 µg/ml) or Stx2B (2 µg/ml) on day 1 and day 2, respectively. Mice were sacrificed, and sera were harvested on day 4 post-injection (pi) for detection of serum creatinine (CRE) and blood urea nitrogen (BUN) with an automatic biochemical analyzer. Serum IL-1β was quantified by ELISA. Kidneys were harvested for histopathological and electron-microscopic examinations. Survival of mice was monitored daily up to 10 days pi.

#### Role of Inflammasomes in Developing Hemolytic Uremic Syndrome *In Vivo*

**Figure 1:**
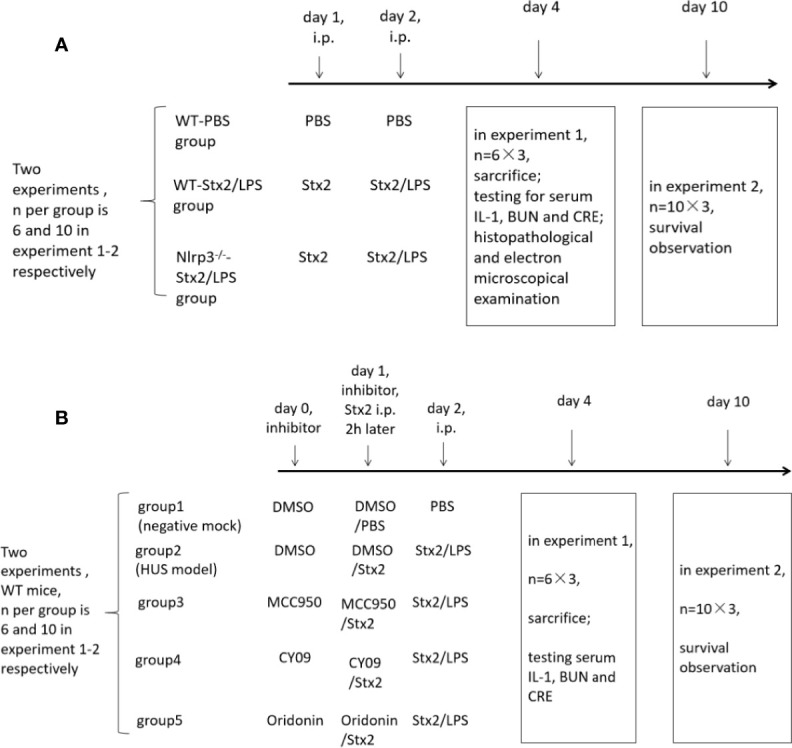
The *in vivo* experiment schedule. To explore the role of the Nlrp3 inflammasome in the development of hemolytic uremic syndrome (HUS) induced by Stx2, two *in vivo* experiments were performed and each experiment included three groups of mice. There were six mice per group in experiment 1 and 10 mice per group in experiment 2 **(A)**; To confirm whether the Nlrp3 inhibitors protect the host from HUS, two *in vivo* experiments were performed and each experiment included five groups of mice. There were six mice per group in experiment 1 and 10 mice per group in experiment 2 **(B)**.

A total of three groups of mice (six mice per group) were used to examine renal function changes in Stx2/LPS treatment. Group 1 (the WT-PBS group) WT mice were injected i.p. with PBS as the negative control; Group 2 (the WT-Stx2/LPS group) WT mice were injected i.p. with Stx2 plus LPS according to the HUS inducement procedure as the positive control; Group 3 (the *Nlrp3*^-/–^Stx2/LPS group) *Nlrp3* deficient mice were treated the same way as the WT-Stx2/LPS group. Sera were harvested on day 4 pi to detect serum CRE, BUN with an automatic biochemical analyzer and IL-1β with ELISA kit (R&D, USA). Kidneys were harvested for histopathological and electron-microscopic examination as detailed in 1.4.5 and 1.4.6). (**Figure 1A**)

Another three groups of mice (10 mice per group) were treated as above and were observed daily for survival up to 10 days pi. (**Figure 1A**)

#### Evaluation of the Role of Nlrp3 Inhibitors in Preventing Hemolytic Uremic Syndrome *In Vivo*

Eight-week-old C57BL/6J WT mice were randomly divided into five groups (six mice per group) and treated as follows. The mock group (negative control) mice were injected i.p. with DMSO on day 0 and day 1, and then with PBS injection on day 1 (2 h after DMSO treatment) and day 2. The HUS model group was injected i.p. with DMSO on day 0 and day 1, and then with Stx2 injection on day 1 (2 h after DMSO treatment) and Stx2 plus LPS on day 2. The three treatment groups were injected i.p. with the Nlrp3 inhibitors diluted in DMSO (50 mg/kg MCC950, 25 mg/kg CY-09; 10 mg/kg Oridonin) on day 0 and day1, and then with Stx2 injection on day 1 (2 h post these inhibitor treatments) and Stx2 plus LPS on day 2 (**Figure 1B**).

These mice were sacrificed and sera were collected on day 4 pi for detecting serum CRE, BUN with an automatic biochemical analyzer and IL-1βwith ELISA kit (R&D, USA) (**Figure 1B**).

The other five groups of mice (10 mice per group) were treated as above and were observed daily for survival up to 10 days pi (**Figure 1B**).

#### Histopathological Examination

On day 4 after Stx2/LPS treatment, the kidneys were harvested and cut into blocks of approximately 1 to 2 mm^3^. The tissue blocks were then fixed in 4% formaldehyde, embedded in paraffin, and sectioned. The sections were stained with hematoxylin-eosin and examined for histopathology in a light microscope.

The kidney injury was scored based on inflammatory cell infiltration and renal tubular injury on a score scaled from 0 to 8 for severity (15, 32). Clinical pathological scoring criteria were used as follows: Inflammatory cell infiltration: 0, no inflammatory cell infiltration; 1, a few small focal inflammatory cell infiltration; 2, scattered focal inflammatory cell infiltration; 3, sizeable inflammatory cell infiltration; 4, diffuse inflammatory cell infiltration.

Tubular injury: 0, normal tubules; 1, less than 25% tubules injured; 2, 25%–49% tubules injured; 3, 50%–74% tubules injured; 4, more than 75% tubules injured.

Multiple (no less than 30) consecutive non-overlapping visual fields under ×100 magnification were examined. The final score was obtained from the average of all visual field scores, which were determined blindly by a clinically experienced pathologist.

#### Electron Microscopy

The ultra-thin sections were stained with uranyl acetate and lead citrate. Photomicrographs were taken at ×6,000 magnification and ×8,000 magnification using a transmission electron microscope (EM) (JEOL-1230, Peabody, MA) in the Laboratory of Electron Microscopy, Peking University First Hospital. At least four glomeruli from each of three mice were examined per group.

### Statistical Analysis

All continuous variables were presented as means ± standard deviation. The univariate analysis of variance (ANOVA) test was used to compare the means of different groups, and the Bonferroni test or Dunnett test, if appropriate, was used for multiple comparisons if their variance homogeneity was assumed. Otherwise, Kruskal-Wallis test was used and the pairwise comparisons also performed if appropriate. The survivals of different groups were plotted with the Kaplan–Meier method, and their multiple comparisons were performed using the log-rank method (pairwise comparison over strata). A αvalue of <0.05 was considered significantly. All statistical analyses were conducted using SPSS 21.0 (SPSS Inc., Chicago, IL).

## Results

### Identification of Recombinant Stx2 Toxin and Subunit B

The recombinant Stx2 and Stx2B were identified by SDS-PAGE and Western blotting (**Figures S1A–D**). The purity of the recombinant proteins was 85%. After 24 h, the Vero cells treated with Stx2 became swollen and round. Most cells died and decomposed within 48 h. The results showed that Stx2 had a dose-dependent cytotoxic effect on Vero cells, and the CD50 of Vero cells for Stx2 was determined to be 10 ng/ml (**Figure S1E**). In contrast, the recombinant Stx2B did not cause cytotoxicity at any dose, which was consistent with the study by Marcato’s et al. (33) (**Figure S1E**).

### *In Vitro* Experiments

#### The Recombinant Stx2 Holotoxin Containing Enzymatically Functional a Unit Is Required for Activation of Caspase-1 and Release of IL-1β and Lactate Dehydrogenase

**Figure 2:**
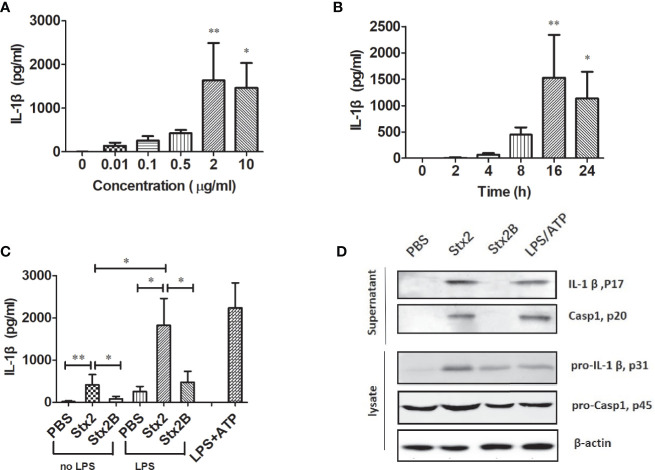
Stx2 triggered IL-1β release *in vitro*. Peritoneal macrophages (PMs) from mice were primed with lipopolysaccharide (LPS) for 4 h before Stx2 treatment (LPS-primed PMs) and were incubated with Stx2 at different doses. The supernatants were harvested for IL-1β detection by ELISA **(A)**. LPS-primed PMs (1 × 10^6^ cells/ml) were treated with Stx2 at different time points. The supernatants were harvested at different time points after Stx2 treatment for the IL-1β detection by ELISA **(B)**. PMs (1×10^6^ cells/ml) were treated with Stx2 (2 μg/ml, 10 μl/well) and Stx2B (2 μg/mL, 10 μl/well) for 16 h with LPS priming in advance or without LPS priming. PBS and LPS plus ATP were used as the negative control and positive control, respectively. The supernatants were collected for IL-1β detection by ELISA **(C)**. Immunoblotting was performed. The culture supernatants were measured for IL-1β p17 and caspase-1 p20; the cell lysates were analyzed for pro-IL-1β p31 and pro-caspase-1 p45 **(D)**. The data in panels **(A–C)** are shown as mean ± standard deviation from three independent experiments. The data in panel **(D)** are obtained from one of two independent experiments. The univariate ANOVA test was used to compare the means of different groups and the Dunnett test was used for their multiple comparison **(A)**. The Kruskal-Wallis test was done to compare the means of different groups with the pairwise comparisons performed **(B, C)**. *p < 0.05, **p < 0.01.

To determine the kinetics of IL-1β secretion induced by Stx2 in PMs, PMs were treated with different doses of Stx2 at different time points. The results showed that PMs treated with 2 µg/ml of Stx2 for 16 h produced the highest level of IL-1β, which was used in the following *in vitro* experiments under optimal conditions (**Figures 2A, B**). To confirm whether the enzymatic A unit of Stx2 is essential to induce IL-1β release, we treated PMs with equal doses of Stx2 holotoxin and the recombinant subunit B of Stx2 (Stx2B) lacking enzymatic activity for 16 h. The results revealed that Stx2 treatment for 16 h induced significantly higher levels of IL-1β compared with the Stx2B treatment in LPS-primed PMs but not in non-primed PMs (**Figure 2C**). Compared with Stx2B, Stx2 induced a significantly higher IL-1β and LDH release but not TNF in PMs, BMDMs, and THP-1 cells (**Figures S2A–C**). To confirm whether Stx2 could activate caspase-1 and induce IL-1β in PMs, we measured the amount of IL-1β (p17) and its immature precursor pro-IL-1β (p31), and the amount of caspase-1 (p20) and its immature precursor pro-caspase-1 (p45) in both supernatants and cell lysates using Western blotting. The results showed that Stx2 induced larger amount of mature IL-1β in the supernatants than subunit B; however, Stx2 and Stx2B induced similar levels of biologically inactive pro-IL-1β in cell lysates. Furthermore, the secretion of the subunit (p20) of caspase-1 was evident in the supernatants of PMs infected with Stx2 or positive control but not in negative control cells or those treated with the Stx2B (**Figures 2D** and **S4A**). Collectively, these data indicate that enzymatically functional Stx2 is required for caspase-1 activation to release the pro-inflammatory cytokine IL-1β.

#### Stx2 Triggers IL-1β and Lactate Dehydrogenase Release Via the Nlrp3 Inflammasome Pathway

**Figure 3:**
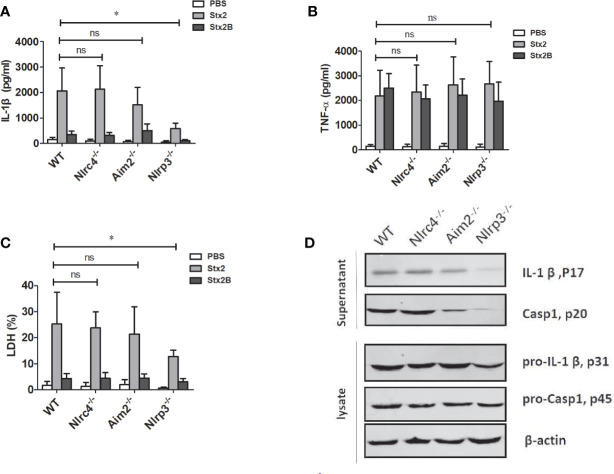
The Stx2-induced IL-1β secretion requires the Nlrp3 inflammasome activation *in vitro*. The Stx2-triggered pro-inflammatory cytokine secretion and lactate dehydrogenase (LDH) leakage were compared among peritoneal macrophages (PMs) derived from wild-type (WT) C57BL/6J or deficient mice (*Nlrc*4^-/-^, *Aim2*^-/-^, *Nlrp3*^-/-^). PMs were primed with LPS for 4 h, followed by Stx2 or Stx2B treatment for 16 h. The supernatants were harvested and assayed for IL-1β **(A)**, TNF-α **(B)**, and LDH **(C)**. The culture supernatant and cell lysates were also harvested to examine the expression of IL-1β (p17), caspase-1 (p20), and their precursors (pro-IL-1β p31, pro-caspase-1 p45) using immunoblotting **(D)**. The data in panels **(A)** to **(C)** are the means ± standard deviations from three independent experiments. The data in panel **(D)** are obtained from one of two independent experiments. The univariate ANOVA test was used to compare the means of different groups and the Dunnett test was used for their multiple comparisons **(A–C)**. *p < 0.05, no significance (ns), p > = 0.05.

To verify the role of inflammasomes in the process of Stx2-induced IL-1β release and cytotoxicity, we treated LPS-primed PMs isolated from WT, *Nlrp3*^-/-^, *Nlrc4*^-/-^ and *Aim*^2-/-^ mice with an equal amount of Stx2 and Stx2B, then examined the release of IL-1β and LDH. We found that Stx2-induced IL-1β and LDH release were significantly reduced in PMs from Nlrp3^-/-^ mice compared with those from WT mice (IL-1β 588.71 ± 206.57 pg/ml vs 2033.28 ± 842.46 pg/ml, p=0.025; LDH 12.84 ± 2.33% vs 25.27 ± 8.13%, p=0.033). IL-1β and LDH release in PM from WT mice were comparable to those from *Nlrc4*^-/-^ and *Aim*^2-/-^ mice. These results suggest that Nlrp3 inflammasome activation may be required in Stx2*-*induced IL-1β production (**Figures 3A, C**).

We also found that TNF-α secretions from all different types of PMs were comparable, suggesting that Nlrp3 inflammasome was dispensable for the production of TNF-α in response to Stx2 (**Figure 3B**).

Consistent with the ELISA results, Stx2 induced higher levels of active and mature IL-1β (p17) and caspase-1 (p20) release in the PMs from WT, *Nlrc4*^-/-^ and *Aim2*^-/-^ mice, but not from the *Nlrp3*^-/-^mice when determined by Western blotting. However, Stx2 induced similar levels of biologically inactive pro-IL-1β and pro-caspase-1 in cell lysates from various cells (**Figures 3D** and **S4B**). Given that Stx2 did not significantly affect the levels of pro-IL-1β in various cells and it induced less IL-1β release in PMs of *Nlrp3*^-/-^ but not PMs from WT, *Nlrc4*^-/-^ and *Aim2*^-/-^ mice, we concluded that the *Nlrp3* inflammasome is required in the process of Stx2*-*induced IL-1β release.

#### Inhibitors Reduce Stx2-Mediated IL-1**β** Release *In Vitro*

**Figure 4:**
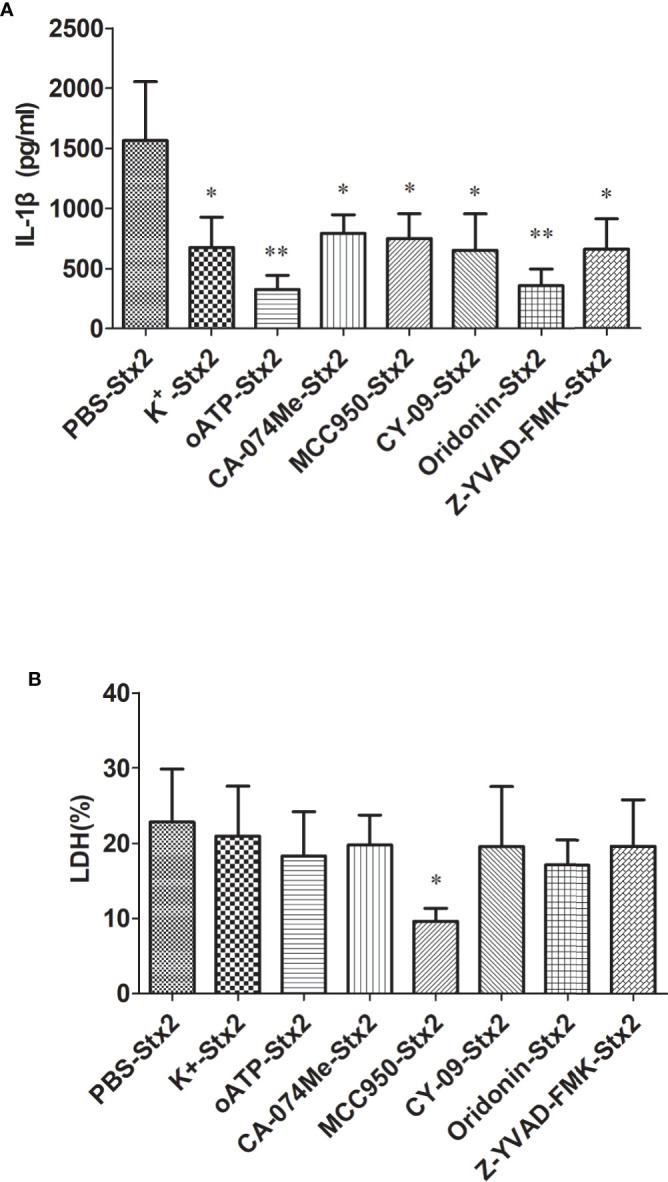
Inhibitors reduce Stx2-Mediated IL-1β release *in vitro*. Primed peritoneal macrophages (PMs) were treated with Stx2 (2 μg/ml, 10 μl/well) for 16 h in the absence (PBS) or presence of the K^+^ efflux blocker (KCl, 50 mM), ATP receptor inhibitor (oxidized ATP, oATP, 500 μM), cathepsin B inhibitor (CA-074Me, 10 μM), Nlrp3 inhibitors (MCC950, 20 μM; CY-09, 5 μM; Oridonin, 20 μM), and caspase-1 inhibitor (Z-YVAD-FMK, 10 μM). IL-1β in the supernatants was measured by ELISA **(A)**, and LDH was assayed using a Cytotox96 Kit **(B)**. The results are represented as the means ± standard deviations of three independent experiments. The univariate ANOVA test was used to compare the means of different groups and the Dunnett test was used for their multiple comparisons **(A, B)**. *p < 0.05; **p < 0.01.

To explore the effects of the inhibitors targeting the Nlrp3 inflammasome pathway to block Stx2-mediated IL-1β release, we pretreated primed-PMs with different inhibitors before Stx2 induction (detailed in Methods section). We found that all these inhibitors could significantly reduce IL-1β release compared to the vehicle control group (p<0.05) with greatest inhibitory effects being observed in cells pretreated with oATP and Oridonin (p<0.01) (**Figure 4A**). LDH release was not significantly attenuated when cells were pretreated with these inhibitors except for MCC950 (**Figure 4B**).

### *In Vivo* Experiments

#### Recombinant Stx2 Holotoxin Together With Lipopolysaccharide Can Induce Hemolytic Uremic Syndrome Mouse Models

Only the mice injected i.p. with Stx2/LPS developed HUS symptoms on day 4 pi as determined by serum CRE and BUN. Serum CRE and BUN were significantly higher in mice treated with Stx2/LPS compared with those treated with PBS, LPS, Stx2, Stx2B, and Stx2B/LPS (**Figures S3A, B**). The serum levels of IL-1β in mice treated with Stx2/LPS were also significantly higher than those seen in Stx2B/LPS and PBS groups (**Figure S3C**). The mice treated with Stx2/LPS began to die on day 3 pi, and all mice died within days 6 pi. The survival rate of mice in the Stx2/LPS group was significantly lower than in the PBS and Stx2B/LPS group (0% vs 100%, p<0.001; 0% vs 90%, p<0.001) (**Figure S3D**).

#### Nlrp3 Inflammasome Activation Contributes to the Development of Stx2-Induced Renal Injuries *In Vivo*

**Figure 5:**
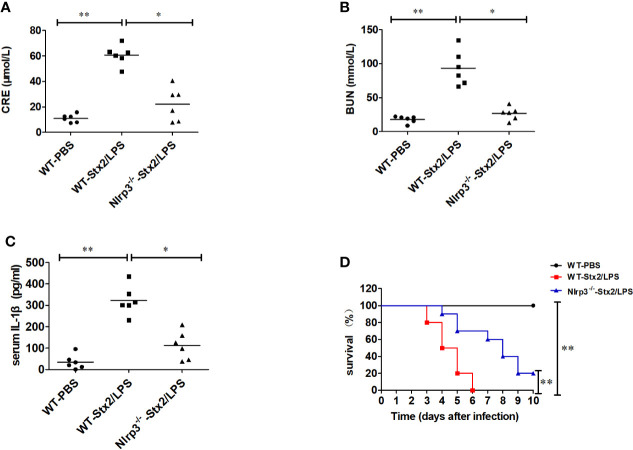
Stx2-mediated IL-1β release requires Nlrp3 inflammasome activation *in vivo*. Three groups of eight-week-old mice (6 mice per group) were chosen to examine renal function changes after Stx2/LPS treatment. Group 1 (WT-PBS group) C57BL/6J WT mice were injected i.p. with PBS as the negative control. Group 2 (WT-Stx2/LPS group) C57BL/6J WT mice were injected i.p. with Stx2 plus lipopolysaccharide (LPS) according to the hemolytic uremic syndrome (HUS) inducement procedure as the positive control, Group 3 (*Nlrp3*^-/–^Stx2/LPS group) *Nlrp3*^-/-^ mice were treated as group 2 was. Sera were harvested on day 4 after injection for detecting serum CRE **(A)**, BUN **(B)**, and serum IL-1β *via* ELISA kit **(C)**. The other similar three groups of mice (treated the same way as above with 10 mice per group) were monitored survival every day up to 6 days after injection **(D)**. The data in panels **(A–C)** are the mean ± standard deviation from three independent experiments. The data in panels **(D)** are obtained from one of two independent experiments. The univariate ANOVA test was used to compare the means of different groups and the Bonferroni test was used for their multiple comparison **(A–C)**. The survivals of different groups of mice were plotted with the Kaplan–Meier method, and their multiple comparisons were performed using the log-rank method (pairwise comparison over strata) **(D)**. *p < 0.05, **p < 0.01.

To confirm the role of Nlrp3 inflammasome in the development of Stx2-induced HUS *in vivo*, we investigated whether Nlrp3 was critical in the pathogenic progress of the kidney using the Stx2-induced HUS mouse model. WT mice treated with Stx2/LPS had significantly higher levels of creatinine (CRE), blood urea nitrogen (BUN), and IL-1β in serum compared with WT mice treated with PBS. However, the levels of CRE, BUN, and IL-1β in serum were significantly alleviated in *Nlrp3*-deficient mice compared with WT mice treated with Stx2/LPS, indicating the involvement of the Nlrp3 pathway in the kidney injuries induced by Stx2/LPS (**Figures 5A–C**). We further observed that WT mice treated with Stx2/LPS all died within 6 days pi and all WT mice treated with PBS survived. The survival within 10 day pi of the *Nlrp3*^-/-^ mice was improved to a certain extent compared with the WT mice after the challenge of Stx2/LPS (20% vs 0%, p<0.001) (**Figure 5D**).

**Figure 6:**
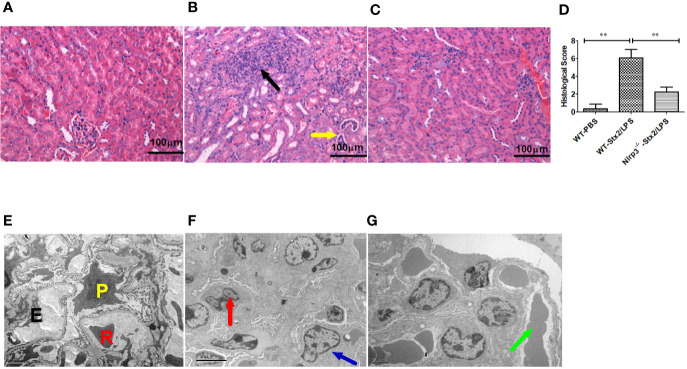
Renal histopathological and glomerular ultrastructural findings in mice 4 days after the challenge. Renal histopathological examination (H-E staining, 100×) are shown in panel **(A–D)**, six mice per group. **(A)** Wild-type (WT)-PBS group (WT mice treated with PBS only). No abnormal findings were observed. **(B)** The WT-Stx2/lipopolysaccharide (LPS) group (WT mice treated by Stx2/LPS). Renal multifocal inflammatory cell (mainly neutrophils) infiltration (black arrow), a small abscess, pyonephrosis (yellow arrow). **(C)** *Nlrp3*^-/-^ -Stx2/LPS group (*Nlrp3*^-/-^ mice treated by Stx2/LPS). Most of the mice showed almost normal renal histopathological findings, and only two mice had mild inflammatory cell infiltration. **(D)** Renal histopathological scores of the above three groups of mice were also presented. Panels **(E–G)** are the representative transmission electron micrographs of glomeruli from the above three groups of mice. **(E)** The WT-PBS group, normal. **(F)** The WT-Stx2/LPS group, neutrophil infiltration (red arrow), endothelial cell swelling, capillary lumen stenosis (blue arrow). **(G)** The *Nlrp3*^-/-^ -Stx2/LPS group, erythrocyte deposition in a capillary lumen in individual mice (green arrow). E, endothelial cell; P, podocyte; R, Red blood cell. Bar = 5 µm, Magnification of ×6,000 in panel **(A)**; Bars = 2 µm, Magnifications of ×8,000 in panel **(B)** through **(F)**. The univariate ANOVA test was used to compare the means of different groups and the Bonferroni test was used for their multiple comparisons **(D)**. *p < 0.05, **p < 0.01.

Histopathological examination of the kidney showed that more infiltration of multifocal inflammatory cells (mainly neutrophils), small abscess, pyonephrosis, renal tubular deterioration and atrophy, lumen dilatation, and leucocytes casts in WT mice treated with Stx2/LPS (WT-Stx2/LPS group) (**Figure 6B**), while there was almost no abnormal findings in the mice from the WT-PBS group (**Figure 6A**) and the *Nlrp3*^-/-^ -Stx2/LPS group (**Figure 6C**). The pathological score of WT mice treated with Stx2/LPS (5.87 ± 1.98) was significantly higher than that of *Nlrp3*^-/-^ mice (2.00 ± 1.48, p = 0.001) and WT mice treated with PBS (0.33 ± 0.40, p <0.001) (**Figure 6D**). The EM results showed swollen glomerular endothelial cells which blocked the capillary lumen, and erythrocyte sedimentation in the capillary lumen and foot process fusion in visceral epithelial cells in WT mice treated with Stx2/LPS (**Figure 6F**). There was no swelling of endothelial cells in the glomeruli, and less erythrocyte sedimentation in capillary lumen occurred in *Nlrp3*^-/-^ mice (**Figure 6G**). Taken together, these data confirmed that the activation of the Nlrp3 pathway contributed to the development of HUS induced by Stx2/LPS.

#### Treatment With Small Molecule Nlrp3 Inhibitors Can Effectively Protect Wild-Type Mice From Renal Injuries on Stx2/Lipopolysaccharide Intraperitoneal Injection

**Figure 7:**
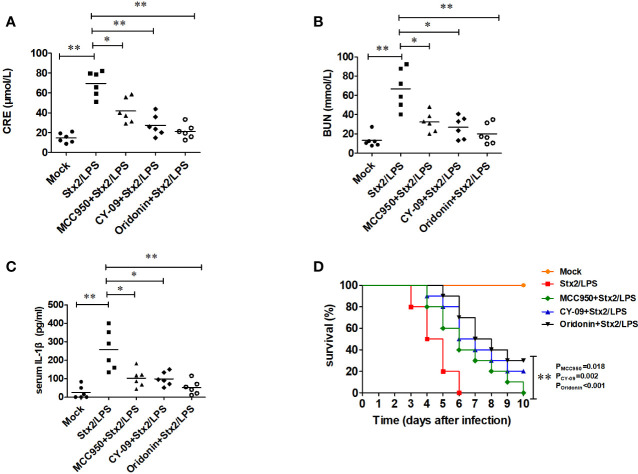
Nlrp3 inhibitors reduce serum IL-1β level and protect renal function in mouse hemolytic uremic syndrome (HUS) models. Eight-week-old C57BL/6J wild-type (WT) mice were randomly divided into five groups, six mice per group. Group 1 (mock group as a negative control) mice were injected i.p. with PBS. Group 2 (Stx2/LPS group as a positive control, namely HUS model group) mice were injected i.p. with Stx2 plus LPS according to the HUS inducement procedure. Group 3 (the MCC950+Stx2/LPS group), group 4 (the CY-09+Stx2/LPS group), and group 5 (Oridonin+ Stx2/LPS group) mice were injected i.p. with three kinds of Nlrp3 inhibitors (MCC950, CY-09, and Oridonin, respectively) before Stx2/LPS treatment. Sera were harvested on day 4 after Stx2/LPS injection for detecting serum CRE **(A)**, BUN **(B)**, and serum IL-1β **(C)**. The other five groups of mice (treated as above but 10 mice per group) were adopted to monitor their survival every day up to 6 days after Stx2/LPS injection **(D)**. The data in panel **(A–C)** are the means ± standard deviation from three independent experiments. The data in panel **(D)** are obtained from one of two independent experiments. The univariate ANOVA test was used to compare the means of different groups and the Dunnett test was used for their multiple comparison **(A)**. The Kruskal-Wallis test was done to compare the means of different groups with the pairwise comparisons performed **(B, C)**. The survivals of different groups of mice were plotted with the Kaplan–Meier method, and their multiple comparisons were performed using the log-rank method (pairwise comparison over strata) **(D)**. *p < 0.05, **p < 0.01.

Compared with the mock PBS-WT group, the mice in Stx2/LPS group as HUS model had a significantly higher level of serum CRE (69.47 ± 12.74 µmol/ml vs 14.63 ± 4.89 µmol/ml, p<0.001), BUN (67.07 ± 20.80 mmol/ml vs 13.24 ± 7.17mmol/ml, p=0.001) and IL-1β (257.13 ± 108.18 pg/ml vs 24.86 ± 34.55 pg/ml, p<0.001) (**Figures 7A–C**). Compared with the HUS mice in Stx2/LPS group, the levels of serum CRE in Oridonin group, MCC950 group, and CY09 group were all significantly decreased (p<0.01 for Oridonin; p<0.05 for MCC950, p<0.01 for CY09) (**Figure 7A**). Similarly, the serum BUN levels in Stx2/LPS group were also significantly higher than those in Oridonin group, MCC950 group, and CY09 group (p<0.01 for Oridonin; p<0.05 for MCC950, p<0.05 for CY09) (**Figure 7B**). Serum IL-1β in Stx2/LPS group were also significantly higher than those in Oridonin group, MCC950 group, and CY09 group (p<0.01 for Oridonin; p<0.05 for MCC950, p<0.05 for CY09) (**Figure 7C**). All 10 mice in Stx2/LPS group died within 6 day pi. Compared with the HUS mice in Stx2/LPS group, the mice pretreated with Oridonin, MCC950 and CY-09 were all protected by postponing the death (P=0.02 for MCC950, p=0.002 for CY09, p<0.001 for Oridonin) although there is not difference in survival rate among them at day 10 pi (**Figure 7D**). While the NLRP3 inhibitors could not protect all HUS mice from eventual death, they could provide partial protection by postponing the death and attenuating inflammation and kidney damage.

## Discussion

Shiga toxin-producing Escherichia coli (STEC) can cause severe HUS, which leads to renal failure and high mortality rates. The key issue in treatment of the STEC infection is reducing renal damage in HUS patients. Stx2 is considered to play an essential role in the development of HUS during STEC infections (8). It has been reported that the subunit B of Stx2 can bind to GB3 receptors on the endothelial cell membrane of glomerular capillary. Then, the subunit A of Stx2 can be transferred into the targeted cells and inhibit protein synthesis through 3’ end of 28 S ribosomal RNA of the 60 S large subunits of ribosome (33, 34). The mouse HUS model was successfully established by Ikeda et al. in 2004 using recombinant Stx2 toxins (17). However, Ikeda et al. observed that the mouse HUS model could not be induced by Stx2 alone unless along with LPS [17]. It is known that systemic inflammation contributes to HUS outcome but the pathogenesis of HUS induced by stx2 is not fully elucidated. Platnich et al. reported that Stx2 could activate the Nlrp3 inflammasome in THP-1 macrophages and increase IL-1β and TNF-α secretion (22). We thus speculated that the Nlrp3 inflammasome activation may play a critical role in the pathogenesis of HUS induced by Stx2. If our hypothesis is confirmed, it would have a promising potential for using Nlrp3 inflammasome inhibitors to treat HUS induced by STEC.

In this study, we observed that the PMs of *Nlrp3*^-/-^ mice produced significantly less IL-1β than PMs of WT mice in response to Stx2/LPS stimulation in the *in vitro* experiments. This result confirms that Stx2 could activate Nlrp3 inflammasome and it is consistent with Platnich’s study (22). To further explore the role of Nlrp3 inflammasome activation in HUS development *in vivo*, we induced a mouse HUS model using Stx2/LPS according to Ikeda’s report using both WT and *Nlrp3*^-/-^mice. We observed that some of the *Nlrp3*^-/-^ mice could be protected from HUS, and their 10-day survival rate was improved significantly compared with WT mice. The kidney pathological examination showed that the kidney of the *Nlrp3*^-/-^mice was less damaged compared with WT mice, which presented obvious renal pathological injuries (massive inflammatory cell infiltration, renal pelvis abscess, tubular deterioration and necrosis). EM scanning of glomeruli revealed that the glomerular ultrastructures of the *Nlrp3*^-/-^ mice were almost normal. In contrast, WT mice were seriously injured (mainly manifested as endothelial cell swelling and podocyte fusion), indicating that the deficiency of the Nlrp3 inflammasome attenuated substantial damage in the kidney. Therefore, our findings provided evidence supporting that the Nlrp3 inflammasome activation induced by Stx2 contributes to the development of HUS, and suggesting that the Nlrp3 inflammasome can be the potential target for HUS therapy.

In line with this, several previous studies have indicated that the Nlrp3 inflammasome activation plays an important role in the pathogenesis of acute kidney injury (35–37). These authors found that Nlrp3 inflammasomes were activated in renal immune cells and renal intrinsic cells (e.g. podocytes, endothelial cells and tubular epithelial cells) in acute kidney injure mouse models, and the inflammasome activation induced by the initial renal injure resulted in leukocyte recruitment (especial *via* IL-1 and IL-18 release), which in turn promoted and amplified the initial renal injure. Van Setten PA et al. (38) also reported that Stx2 could activate Nlrp3 inflammasome and release IL-1β, and IL-1β could in turn upregulate the expression of the Gb3 receptors on cell membranes of target organs such as the kidney, thus improving the sensitivity of the host to the depurination toxicity of Stx2. The above-mentioned studies were in agreement with our findings in the current study. However, the question remains to be answered is how the Nlrp3 inflammasome activation induced by Stx2 further causes HUS, by increasing the renal sensitivity to the Stx2 subunit A *via* upregulating the expression of Gb3 receptor on renal cell membrane, or by excessive inflammatory response *via* recruiting leukocytes to the renal local tissue. This calls for future studies to determine.

To investigate the therapeutic effects of the available inhibitors of Nlrp3 inflammasome activation, we first found that IL-1β secretion was significantly decreased if WT PMs were pretreated with any of three types of Nlrp3 inhibitors before Stx2/LPS treatment *in vitro*. These results confirmed the inhibitory effect of these small molecule inhibitors on the activation of Nlrp3 inflammasome activation *in vitro*. Furthermore, we examined the effect of these small molecule inhibitors (39–41) on preventing mouse HUS development *in vivo* experiments. The results showed that the 6-day survival rate of the mice pretreated with these inhibitors was higher compared to the positive control group pretreated with DMSO without inhibitors. Among these small molecule inhibitors, Oridonin presented the best protecting effect. The serum IL-1β of the Oridonin group was significantly reduced compared to that of the positive control group, indicating that the activation of the Nlrp3 inflammasome pathway was blocked effectively by Oridonin. The serum CRE and BUN levels in the Oridonin group mice were also significantly lower than those of the positive control group, indicating that pretreatment of Oridonin could attenuate the renal injures in the development of HUS. Our study confirm that Oridonin contributes to protecting mice from developing HUS when they are challenged with Stx2/LPS by inhibiting the activation of Nlrp3 inflammasome. Given that Oridonin is an approved drug with reasonable safety, it may act as adjunctive treatment for HUS.

In conclusion, the activation of the Nlrp3 inflammasome induced by Stx2 plays a critical role in the development of HUS. Oridonin, a small molecule inhibitor targeting Nlrp3 inflammasome, can specifically suppress the activation of Nlrp3 inflammasome, alleviate renal injures and improve animal survival in HUS development. Nlrp3 inhibitors may be a promising adjunctive drug for the prevention and treatment of HUS.

## Data Availability Statement

The original contributions presented in the study are included in the article/**Supplementary Material**; further inquiries can be directed to the corresponding authors.

## Ethics Statement

The animal study was reviewed and approved by the Laboratory Animal Welfare and Ethics Committee of the National Institute for Communicable Disease Control and Prevention, Chinese Center for Disease Prevention and Control.

## Author Contributions

LS and ZR conceptualized the experiments. LS, XL, YH, and YX conducted the experiments. LS analyzed the data. LS and ZR wrote the paper. All authors contributed to the article and approved the submitted version.

## Funding

This work was supported by grants from the National Natural Science Foundation of China (No. 81371761 to ZR and 31170868 to GM) and the Ministry of Science and Technology of China (Grant No. 2018ZX10301403-003-003 to SL and 2018ZX10712-001-006 and 2018ZX10305409-003-001 to ZR).

## Conflict of Interest

The authors declare that the research was conducted in the absence of any commercial or financial relationships that could be construed as a potential conflict of interest.
