# Supplementary material for: Activation of the Nlrp3 Inflammasome Contributes to Shiga Toxin-Induced Hemolytic Uremic Syndrome in a Mouse Model
Source: Front Immunol. 2021 Jan 21;11:619096. doi: 10.3389/fimmu.2020.619096 (PMC7859089; doi:10.3389/fimmu.2020.619096)
Supplement: Supplementary file 1 [file DataSheet_1.docx]

**Figure S1: Identification, Vero cell cytotoxicity of recombinant Stx2 protein and its subunit B (Stx2B).**

SDS-PAGE analysis (A and B): lane 1 and lane 4, BSA (1 μg); lane 2 and lane 5, Marker; lane 3, purified Shiga toxin 2 (Stx2, 1 μg); lane 6, purified subunit B of Stx2 (Stx2B, 1 μg)

Western blotting (C and D): lane 2 and lane 3, Marker; lane 1, purified Stx2 (C); lane 4, Stx2B (D)

Vero cell mortality was examined by the LDH method. Vero cells were incubated with various doses of Stx2 from 1 pg to 1 μg, Stx2B as a control. The LDH leakage of Vero cells treated was detected. LDH, lactate dehydrogenase (E).

**Figure S2: Choice of three macrophages.**

Three types of macrophages, including human monocytic THP-1 cells, mouse PMs and BMDMs, were infected with Stx2 or its subunit B (Stx2B) for 16 h after LPS priming for 4 h. The supernatants were harvested for measuring IL-1β (A), TNF-α (B), and LDH (C). The univariate ANOVA test was used to compare the means of Stx2 and Stx2B groups and the Bonferroni test was used for their multiple comparison (A, B, C), **p < 0.01.

**Figure S3: Mouse HUS model induced by Stx2/LPS.**

Eight-week-old C57BL/6J WT mice were randomly divided into six groups, six mice per group. Group 1 (group PBS) mice were injected intraperitoneally (i.p.) with PBS, group 2 (group LPS) with LPS, group 3 (group Stx2) with Stx2, group 4 (group Stx2/LPS) with Stx2 plus LPS, group 5 (group Stx2B) with subunit B and group 6 (group Stx2B/LPS) with subunit B plus LPS. Sera were harvested on day four after injection for detecting serum CRE (A), BUN (B) and IL-1β.

The other three groups of mice (treated as above but 10 mice per group), including group PBS, group Stx2/LPS, and group Stx2B/LPS, were examined for their survival every day up to six days after injection (D).

The data in panels A to C are the mean ± standard deviation from three independent experiments. The data in panels D are obtained from one of two independent experiments.

The Kruskal-Wallis test was done to compare the means of different groups with the pairwise comparisons performed. The survivals of different groups of mice were plotted with the Kaplan–Meier method, and their multiple comparisons were performed using the log-rank method (pairwise comparison over strata). *p < 0.05, **p < 0.01.

**Figure S4: Quantitative analyses f**or the Western Blotting experiments.

The Western Blotting electrophoresis graphs were quantified by the Image J software. A) Quantitative analyses for panel D in Figure 2 (Stx2 triggered IL-1β release in vitro); B) Quantitative analyses for panel D in Figure 3 (The Stx2-induced IL-1β secretion requires the Nlrp3 inflammasome activation in vitro).
