# Supplementary figures and images for: Activation of the Nlrp3 Inflammasome Contributes to Shiga Toxin-Induced Hemolytic Uremic Syndrome in a Mouse Model
Source: Front Immunol. 2021 Jan 21;11:619096. doi: 10.3389/fimmu.2020.619096 (PMC7859089; doi:10.3389/fimmu.2020.619096)

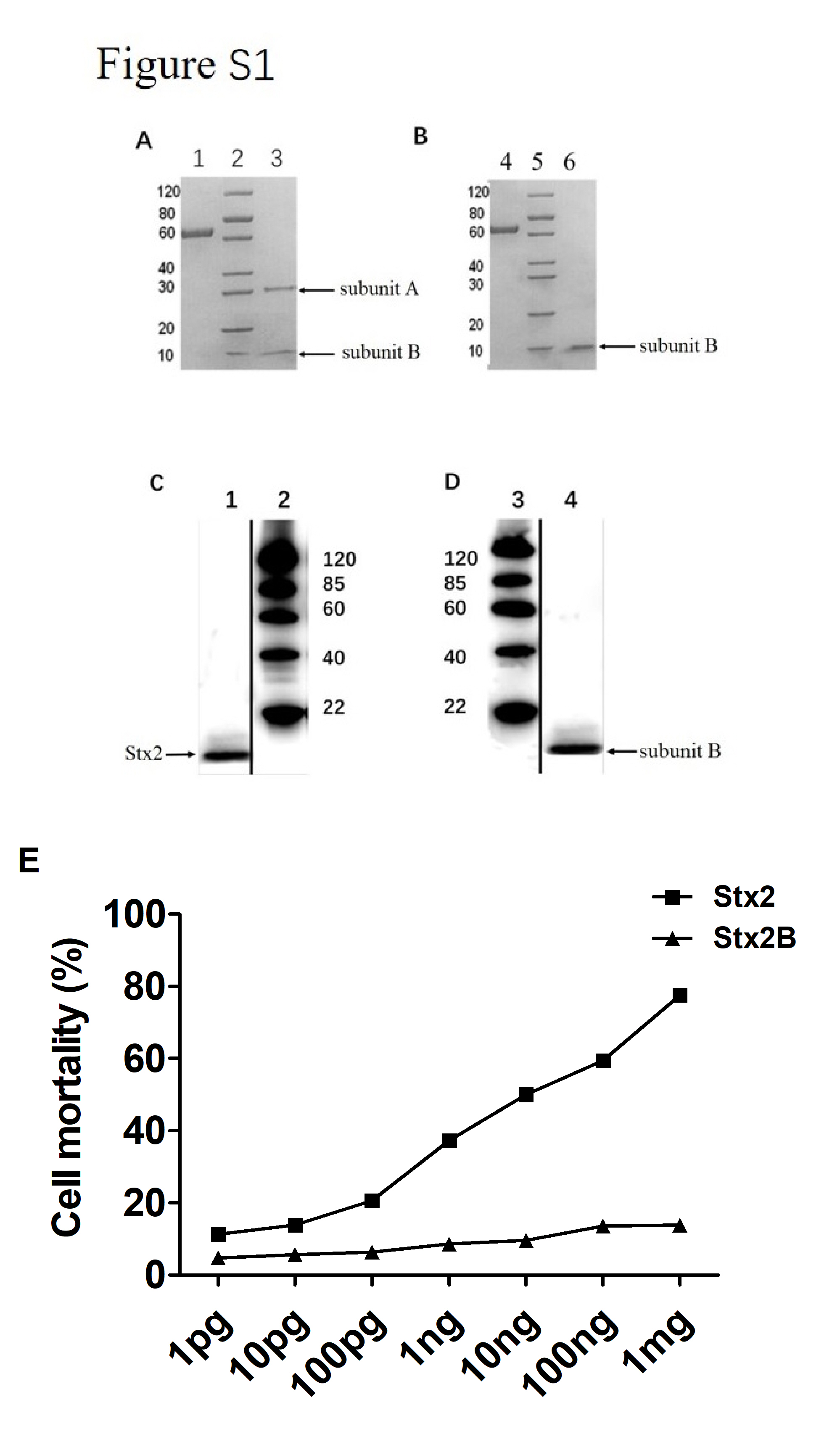

Supplement: Supplementary file 2 [file Image_1.tiff]

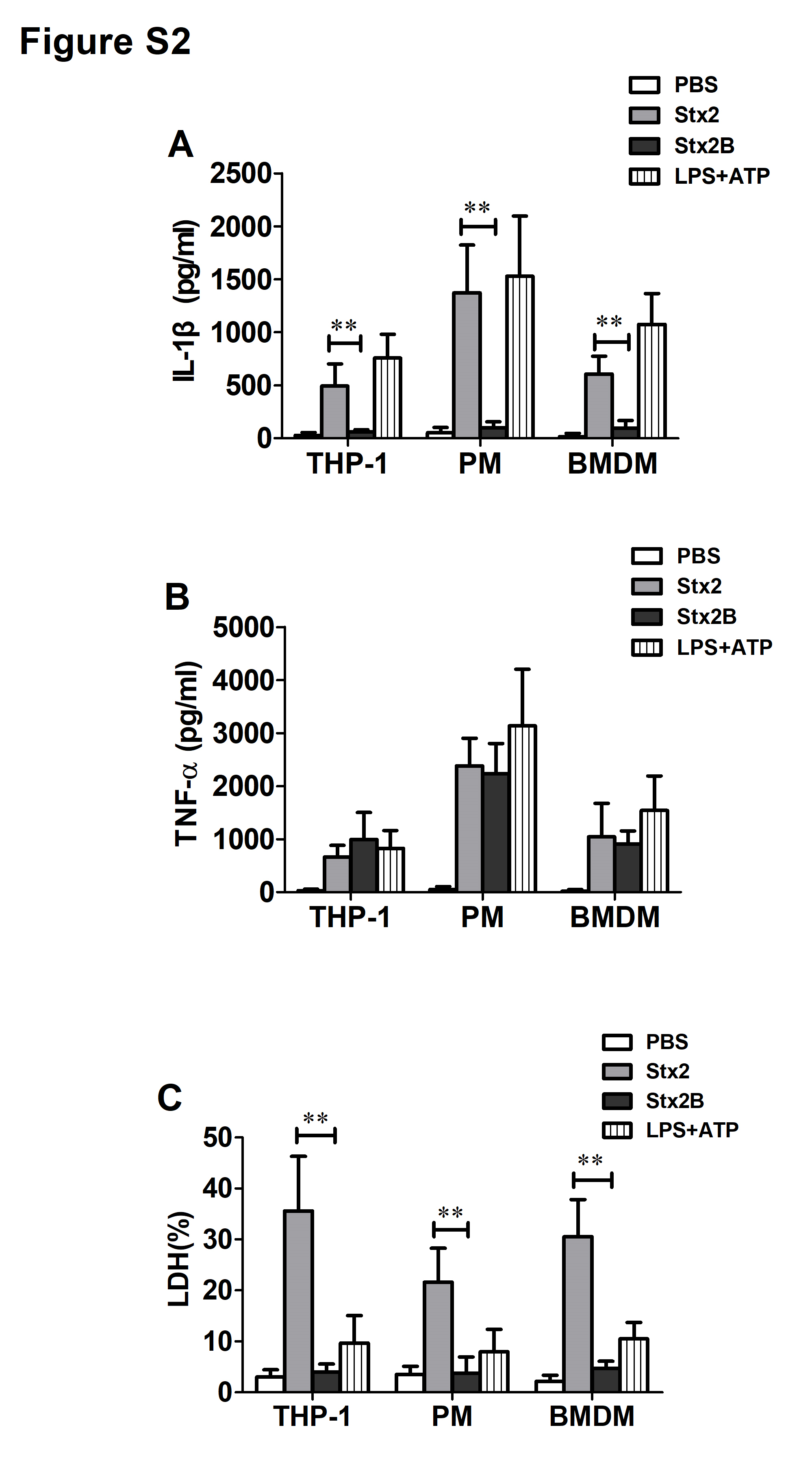

Supplement: Supplementary file 3 [file Image_2.tiff]

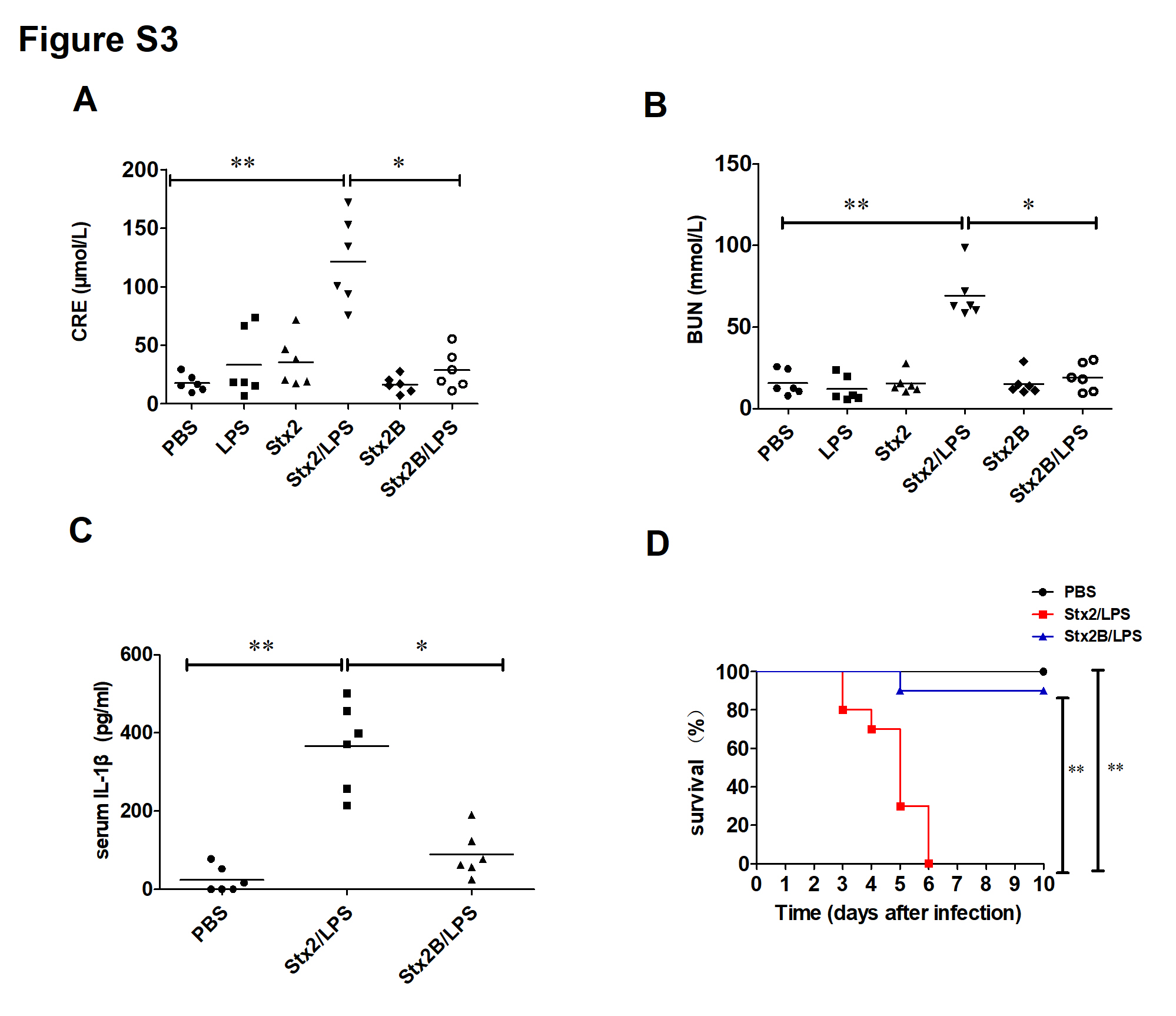

Supplement: Supplementary file 4 [file Image_3.tiff]

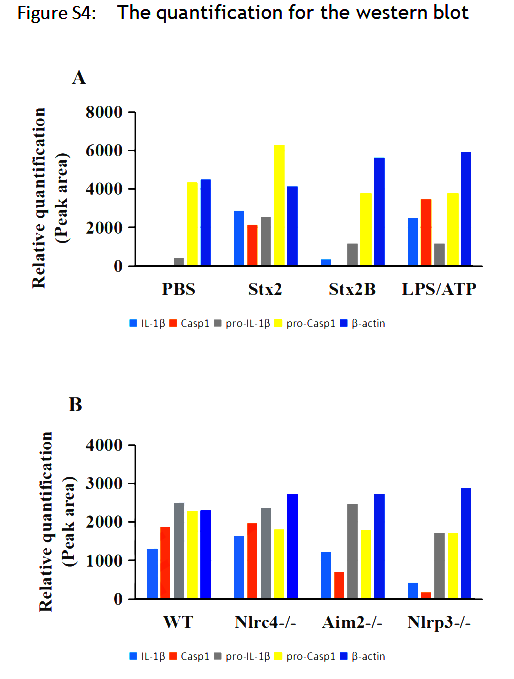

Supplement: Supplementary file 5 [file Image_4.tif]
